# Hydrophilic and lipophilic statin use and risk of hearing loss in hyperlipidemia using a Common Data Model: multicenter cohort study

**DOI:** 10.1038/s41598-023-39316-x

**Published:** 2023-07-31

**Authors:** Insik Song, Minjin Kim, Hangseok Choi, Jeong Hwan Kim, Kang Hyeon Lim, Hee Soo Yoon, Yoon Chan Rah, Euyhyun Park, Gi Jung Im, Jae-Jun Song, Sung-Won Chae, June Choi

**Affiliations:** 1 Department of Otorhinolaryngology-Head and Neck Surgery, Ansan Hospital, Korea University College of Medicine, Ansan, Republic of Korea; 2 Department of Biostatistics, Korea University College of Medicine, Seoul, Republic of Korea; 3 Biomedical Research Center, Korea University Ansan Hospital, Ansan, Republic of Korea; 4 Department of Medical Informatics, Korea University College of Medicine, Seoul, Republic of Korea; 5 Department of Otorhinolaryngology-Head and Neck Surgery, Anam Hospital, Korea University College of Medicine, Seoul, Republic of Korea; 6 Department of Otorhinolaryngology-Head and Neck Surgery, Guro Hospital, Korea University College of Medicine, Seoul, Republic of Korea

**Keywords:** Epidemiology, Outcomes research

## Abstract

Hearing impairment, the third largest health burden worldwide, currently lacks definitive treatments or preventive drugs. This study compared the effects of hydrophilic and lipophilic statin on hearing loss using a common database model. This retrospective multicenter study was conducted in three hospitals in South Korea (Anam, Guro, Ansan). We enrolled patients with hyperlipidemia with an initial hearing loss diagnosis. Data were collected during January 1, 2022–December 31, 2021 using the Observational Health Data Science and Informatics open-source software and Common Data Model database. The primary outcome was the occurrence of first-time hearing loss following a hyperlipidemia diagnosis, as documented in the Common Data Model cohort database. The measures of interest were hearing loss risk between hydrophilic and lipophilic statin use. Variables were compared using propensity score matching, Cox proportional regression, and meta-analysis. Among 37,322 patients with hyperlipidemia, 13,751 (7669 men and 6082 women) and 23,631 (11,390 men and 12,241 women) were treated with hydrophilic and lipophilic statins, respectively. After propensity score matching, according to the Kaplan–Meier curve, hearing loss risk did not significantly differ among the hospitals. The hazard ratio (HR) of the male patients from Anam (0.29, [95% confidence interval (CI), 0.05–1.51]), Guro (HR, 0.56, [95% CI 0.18–1.71]), and Ansan (hazard ratio, 0.29, [95% CI 0.05–1.51]) hospitals were analyzed using Cox proportional regression. Overall effect size (HR, 0.40, [95% CI 0.18–0.91]) was estimated using meta-analysis, which indicated that hearing loss risk among hydrophilic statin users was less than that among lipophilic statin users and was statistically significant. Men in the hydrophilic statin group had a lower risk of hearing impairment than those in the lipophilic statin group.

## Introduction

Hearing impairment is a pervasive disorder, and its incidence increases with age and is the third largest health burden globally^1^. For decades, efforts to develop medical interventions for preventing or mitigating hearing loss have been ongoing. Several drugs and approaches have been studied in vitro, in animals, and in clinical trials. While some have looked promising, to date, no drug has been approved by the U.S. Food and Drug Administration (FDA) for treating hearing loss^2–5^.

Cardiovascular damage and neurodegeneration are known risk factors for hearing loss^6–8^. Statins, which are commonly used to treat cardiovascular and neurological disorders, may also be effective for treating hearing impairment^5,9^. Statins are a class of drugs that are commonly used for the treating hyperlipidemia and for preventing heart attack and stroke. Statins are classified into two categories: hydrophilic and lipophilic. Lipophilic statins, which have a high permeability to adipose tissue, have been shown to attenuate adipocyte maturation. In contrast, hydrophilic statins do not have this effect^10^. Hydrophilic statins have been found to lower serum adiponectin and C-reactive protein levels. Some previous studies have suggested the potential of hydrophilic statins to yield a more pronounced impact on the occurrence of hearing loss when contrasted with their lipophilic counterparts^9,10^. Other studies have investigated the association between statin intake and the risk of hearing loss and found that the association may be influenced by sex^5,11,12^.

The Common Data Model (CDM) cohort study involved a correlation analysis of specific topics through a model that defines different large-scale clinical data in each hospital with the same standards of structure and meaning. This approach allows for easy gathering and utilization of data, making it popular in multicenter research^13^.

In this multicenter study, we compared the impact of hydrophilic and lipophilic statins on hearing loss by utilizing a common database model. Additionally, we specifically examined the sex-related effect of statins on hearing loss.

## Materials and methods, study design, and data set

This multicenter retrospective cohort study was conducted at three hospitals (Korea University Anam, Guro, and Ansan hospitals) from January 1, 2002 to December 31, 2021. It used the Observational Health Data Sciences and Informatics (OHDSI)^14^ open-source software and the CDM database^15^. The OHDSI network is an international collaboration that aims to develop a data-sharing system^14,16^ by applying open-source data analytics to a large number of health databases^17^.

### Patient selection

Patients (aged 40–80 years) with hyperlipidemia, identified using the International Classification of Diseases, Tenth Revision, Clinical Modification (ICD-10-CM) diagnosis code E78.2, were selected. These were identified using a concept ID code in the CDM database and their details, including hearing loss, are provided in Table S1. Among the patients with hyperlipidemia, only those with a first diagnosis of hearing loss were included, and patients previously diagnosed with hearing loss were excluded. The patients were divided into two groups: hydrophilic and lipophilic statins users. Furthermore, the study excluded participants who had an outcome date within 6 months of starting treatment with hydrophilic statins and had been previously prescribed lipophilic statins. Similarly, participants who had an outcome date within 6 months of starting treatment with lipophilic statins and had previously been prescribed hydrophilic statins were also excluded. Moreover, we divided patients based on their sex to understand its effect separately.

### Outcomes and other variables

The primary outcome was the first occurrence of hearing loss followed by a diagnosis of hyperlipidemia. Hearing loss includes sensorineural hearing loss, sensory hearing loss, neural hearing loss, noise-induced hearing loss, and sudden hearing loss. The classification and identification were based on the systematic nomenclature of medicine (SNOMED), which is a standardized vocabulary for diagnostic codes. To eliminate confounding factors while setting covariates, we excluded patients taking aminoglycosides^17,18^ and platinum compounds^19,20^, as these may impact hyperlipidemia regardless of the type of statins used.

### Statistical analyses

To control the selection bias, we used propensity score (PS) matching to balance the covariates between the hydrophilic statin and lipophilic statin groups. However, in this study, the preference score was used after suitable adjustments. The equation of the preference score is as follows:

$$\mathrm{ln}\left(\frac{Preference\, score}{1-Preference\, score}\right)=\mathrm{ln}\left(\frac{Propensity\, score}{1-Propensity\, score}\right)-\mathrm{ln}\left(\frac{Proportion}{1-Proportion}\right)$$

where *Proportion* is the proportion of participants receiving the treatment.

We performed a matched group analysis using 1:1 propensity matching with a 0.25 caliper. Additionally, we created a table for baseline characteristics and applied Cox proportional regression to calculate the hazard ratio (HR), which was used to investigate the association between statin intake and hearing loss. The Kaplan–Meier cumulative survival plot was used to compare the difference before and after PS matching for each hospital. The HR was calculated using Cox proportional regression and the overall correlations were evaluated using a meta-analysis. Statistical approaches (Cochran’s Q test and I^2^ value test) were used to test for heterogeneity in the meta-analysis^21^. All statistical analyses were conducted using R 4.1.3 (http://www.R-project.org) and the OHDSI Cohort Method R package^22^. Meta-analyses of the random-effects model were performed using meta for the R package.

We used seven negative control outcomes to check systematic errors. These included acute bronchitis, essential hypertension, gastro-esophageal reflux disease with esophagitis, gingival and periodontal disease, and spinal stenosis. The selection criteria for negative controls were outcomes that were not anticipated to be affected using either hydrophilic or lipophilic statins. Therefore, it was assumed that their HR would be 1. Calibration plots were generated, and the calibrated *p*-value and credible interval for negative controls were used to visualize uncertainty.

### Ethical approval

This study was approved by the ethics committee of each hospital (ANAM IRB No.: 2022AN0376, GURO IRB No.: 2022GR0368, ANSAN IRB No.: 2022AS0182), and the requirement for informed consent was waived by the ethics committee along with the Institutional Review Board of Korea University Anam, Guro, and Ansan hospitals. All methods were performed in accordance with the relevant guidelines and regulations.

## Results

### Patient selection and clinical characteristics

**Figure 1:**
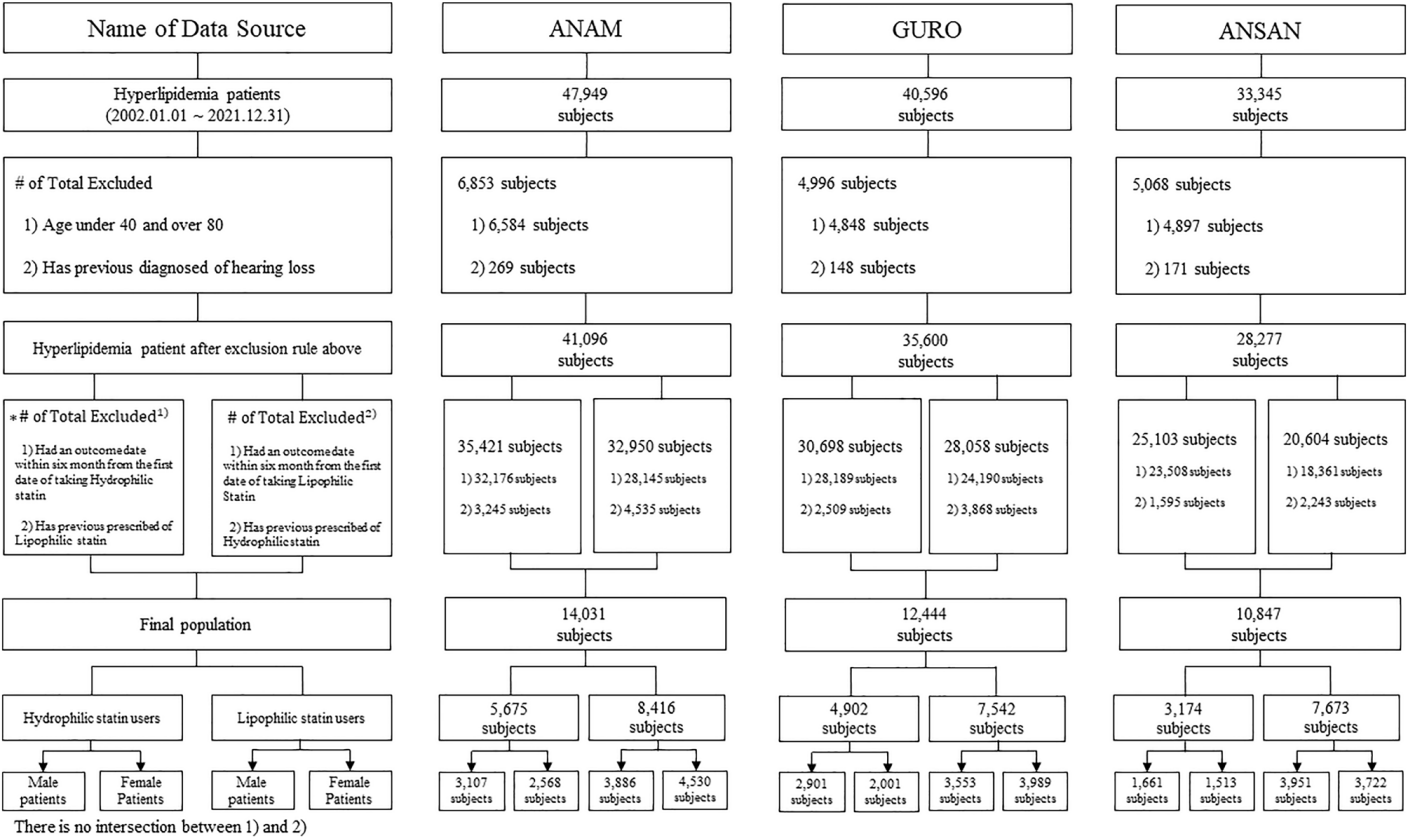
Flowchart of the study participants in the Common Data Model (CDM) network.

This study included 37,322 patients with hyperlipidemia from three hospitals. Among these participants, 13,751 (7669 men and 6082 women) and 23,631 (11,390 male and 12,241 female) patients used hydrophilic and lipophilic statins, respectively. Figure 1 presents a patient flowchart of the study. Detailed information about the number of cases and incidence rate per 1000 person-years from each hospital is presented in Table S2.

Table S2 illustrates the incidence rate per 1000 person-years of hearing loss after propensity score matching of patients from the three hospitals. According to Table S2, after matching, the 1000 person-year incidence rates (IR) among lipophilic statin users (IR = 2.48) were slightly lower than that among hydrophilic statin users (IR = 3.12) among patients from the Guro hospital. However, the incidence rates of patients from the Anam (IR = 3.33–3.89) and Ansan (IR = 3.30–3.33) hospitals showed an opposite trend. These results do not represent a significant difference between both groups because the time at risk is different. Detailed information about the incidence of hearing loss according to sex is described in Table S3. The median follow-up time was 1303, 1402, and 1038 days for patients from the Anam, Guro, and Ansan hospitals, respectively.

The aggregated baseline characteristics of hydrophilic and lipophilic statin users from each hospital before and after PS matching are described in Table 1. More than 50% of the patients in both groups were aged between 50 and 74 years, and very few patients were over 80 years old. Approximately 20% of the proportion of both statin users had diabetes mellitus, while approximately 42% had hypertensive disorder. Additionally, the proportion of smokers was extremely low among statin users in all three hospitals.

**Table 1:** Study participants’ selected baseline characteristics before and after propensity score matching.

Characteristics	ANAM					
	Before matching		Standard difference	After matching		Standard difference
	Hydrophilic users (n = 5675)	Lipophilic users (n = 8416)		Hydrophilic users (n = 2510)	Lipophilic users (n = 2510)	
	No. (%)	No. (%)		No. (%)	No. (%)	
Age (years)						
40–44	316 (5.6)	426 (5.1)	0.02	148 (5.9)	141 (5.6)	0.01
45–49	458 (8.1)	655 (7.8)	0.01	205 (8.2)	224 (8.9)	− 0.03
50–54	786 (13.9)	1100 (13.1)	0.02	344 (13.7)	331 (13.2)	0.02
55–59	974 (17.2)	1371 (16.3)	0.02	445 (17.7)	432 (17.2)	0.01
60–64	986 (17.4)	1488 (17.7)	− 0.01	434 (17.3)	450 (17.9)	− 0.02
65–69	856 (15.1)	1288 (15.3)	− 0.01	356 (14.2)	355 (14.1)	0.00
70–74	669 (11.8)	1107 (13.2)	− 0.04	287 (11.4)	292 (11.6)	− 0.01
75–79	527 (9.3)	861 (10.2)	− 0.03	241 (9.6)	251 (10)	− 0.01
≥ 80	102 (1.8)	120 (1.4)	0.03	50 (2)	34 (1.4)	0.05
Male	3107 (54.7)	3886 (46.2)	0.17	1222 (48.7)	1230 (49)	− 0.01
Female	2568 (45.3)	4530 (53.8)	− 0.17	1288 (51.3)	1280 (51)	0.01
Diabetes mellitus	1012 (17.8)	1865 (22.2)	− 0.11	494 (19.7)	458 (18.2)	0.04
Hypertensive disorder	2576 (45.4)	3458 (41.1)	0.09	1025 (40.8)	1076 (42.9)	− 0.04
Smoker	1013 (17.9)	1814 (21.6)	− 0.09	420 (16.7)	422 (16.8)	0.00

Characteristics	GURO					
	Before matching		Standard Difference	After matching		Standard difference
	Hydrophilic users (n = 4902)	Lipophilic users (n = 7542)		Hydrophilic users (n = 2182)	Lipophilic users (n = 2182)	
	No. (%)	No. (%)		No. (%)	No. (%)	
Age (years)						
40–44	279 (5.7)	388 (5.1)	0.02	127 (5.8)	131 (6)	− 0.01
45–49	434 (8.9)	597 (7.9)	0.03	194 (8.9)	197 (9)	0.00
50–54	676 (13.8)	1029 (13.6)	0.00	294 (13.5)	308 (14.1)	− 0.02
55–59	883 (18)	1342 (17.8)	0.01	402 (18.4)	395 (18.1)	0.01
60–64	952 (19.4)	1374 (18.2)	0.03	418 (19.2)	367 (16.8)	0.06
65–69	719 (14.7)	1164 (15.4)	− 0.02	323 (14.8)	346 (15.9)	− 0.03
70–74	517 (10.5)	934 (12.4)	− 0.06	246 (11.3)	246 (11.3)	0.00
75–79	383 (7.8)	625 (8.3)	− 0.02	151 (6.9)	160 (7.3)	− 0.02
≥ 80	59 (1.2)	89 (1.2)	0.00	27 (1.2)	32 (1.5)	− 0.02
Male	2901 (59.2)	3553 (47.1)	0.24	1110 (50.9)	1162 (53.3)	− 0.05
Female	2001 (40.8)	3989 (52.9)	− 0.24	1072 (49.1)	1020 (46.7)	0.05
Diabetes mellitus	951 (19.4)	1790 (23.7)	− 0.11	456 (20.9)	432 (19.8)	0.03
Hypertensive disorder	2115 (43.1)	3520 (46.7)	− 0.07	938 (43)	887 (40.7)	0.05
Smoker	957 (19.5)	967 (12.8)	0.18	270 (12.4)	283 (13)	− 0.02

Characteristics	ANSAN					
	Before matching		Standard Difference	After matching		Standard Difference
	Hydrophilic users (n = 3174)	Lipophilic users (n = 7673)		Hydrophilic users (n = 1655)	Lipophilic users (n = 1655)	
	No. (%)	No. (%)		No. (%)	No. (%)	
Age (years)						
40–44	233 (7.3)	700 (9.1)	− 0.06	130 (7.9)	140 (8.5)	233 (7.3)
45–49	404 (12.7)	975 (12.7)	0.00	210 (12.7)	177 (10.7)	404 (12.7)
50–54	615 (19.4)	1313 (17.1)	0.06	312 (18.9)	310 (18.7)	615 (19.4)
55–59	614 (19.3)	1397 (18.2)	0.03	271 (16.4)	292 (17.6)	614 (19.3)
60–64	487 (15.3)	1142 (14.9)	0.01	249 (15)	265 (16)	487 (15.3)
65–69	354 (11.2)	811 (10.6)	0.02	201 (12.1)	197 (11.9)	354 (11.2)
70–74	258 (8.1)	674 (8.8)	− 0.02	155 (9.4)	146 (8.8)	258 (8.1)
75–79	179 (5.6)	579 (7.5)	− 0.08	111 (6.7)	113 (6.8)	179 (5.6)
≥ 80	30 (0.9)	82 (1.1)	− 0.01	16 (1)	15 (0.9)	30 (0.9)
Male	1661 (52.3)	3951 (51.5)	0.02	815 (49.2)	814 (49.2)	1661 (52.3)
Female	1513 (47.7)	3722 (48.5)	− 0.02	840 (50.8)	841 (50.8)	1513 (47.7)
Diabetes mellitus	724 (22.8)	1661 (21.6)	0.03	446 (26.9)	461 (27.9)	724 (22.8)
Hypertensive disorder	1415 (44.6)	3177 (41.4)	0.06	771 (46.6)	800 (48.3)	1415 (44.6)
Smoker	419 (13.2)	1358 (17.7)	− 0.12	235 (14.2)	194 (11.7)	419 (13.2)

**Figure 2:**
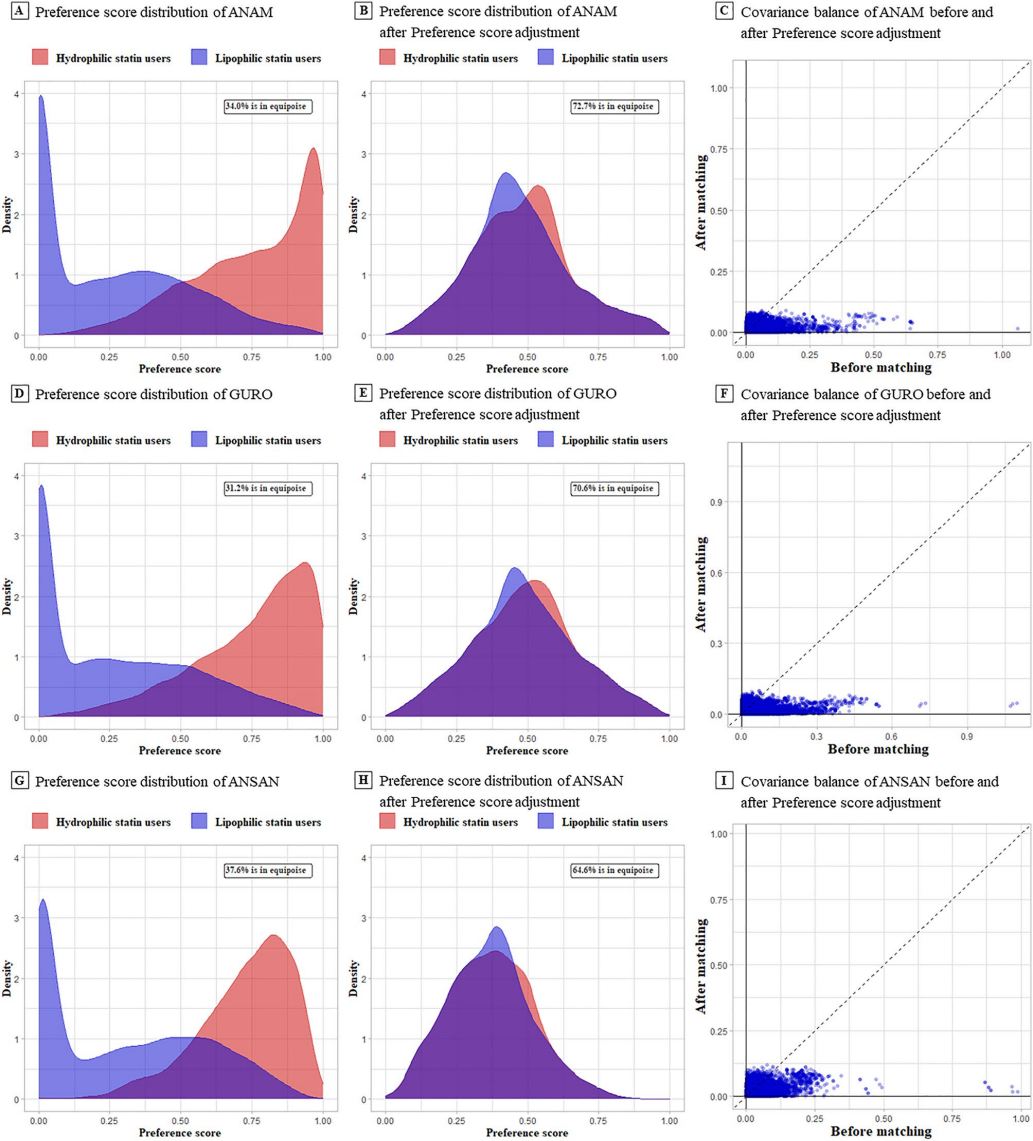
Preference score distribution and covariate balancing before and after matching of the three hospitals. (**A**), (**D**), and (**G**) are the preference score distributions before propensity score (PS) matching. It shows that the overlapping areas are only between 31.2 and 37.6%, indicating that adjustment is necessary. (**B**), (**E**), and (**H**) display similar preference score distributions as (**A**), (**D**), and (**G**) but with an approximately sufficient overlap after PS matching. This demonstrates the perfect success of the adjustment in achieving balance. (**C**), (**F**), and (**I**) illustrate the covariate balance of the three hospitals before and after preference score matching. Each blue dot contains the standardized difference means for a single covariate before and after PS matching. The figure represents poor balance before but well-balanced after matching, with all covariates (28,898 for Anam, 26,341 for Guro, and 26,070 for Ansan) under 0.1 and most under 0.05. This figure illustrates that the adjustment successfully balanced all measured variables and that the two cohorts are in fact similar in all measured aspects.

Figure 2 presents the transformed preference score distribution into a PS adjustment for each hospital. Figure 2A,D and G show the preference score distribution before matching, and Fig. 2B,E and H present the PS distribution after matching. These graphs illustrate perfect overlapping after PS adjustment, which actively demonstrates that the PS distribution of each hospital was successful in balancing covariates. The percentage of the patients in equipoise for the propensity model can be computed. In the preference score distribution after matching, the proportions overlapped according to equipoise (72.7%, 70.6%, and 64.6% for Anam, Guro, and Ansan hospital patient groups, respectively). Figure 2C,F and I show the graphs of the covariate balance before and after preference score matching. Each dot represents the standardized difference of the means for a single covariate before and after PS matching^23^. The X-coordinates of each graph show the absolute value of the standardized difference of the mean before PS matching, and the Y-coordinates represent the value after PS matching. Each dot represents a covariate and perfect balance after matching, according to most dots in the Y-coordinate located below 0.1. Among these patients, the covariates were well-balanced in each data source. The types of covariates included demographics (i.e., sex, 5-year bands age group), conditions in the prior 30 days and 365 days, and aggregation of the condition in the SNOMED CT. Aggregation of drug is ingredient and Anatomical Therapeutic Chemical Classification System (ATC class). The results of the preference distribution into a PS adjustment for each hospital by sex are shown in Fig. S2.

### Risk of hearing loss associated with statin use

**Figure 3:**
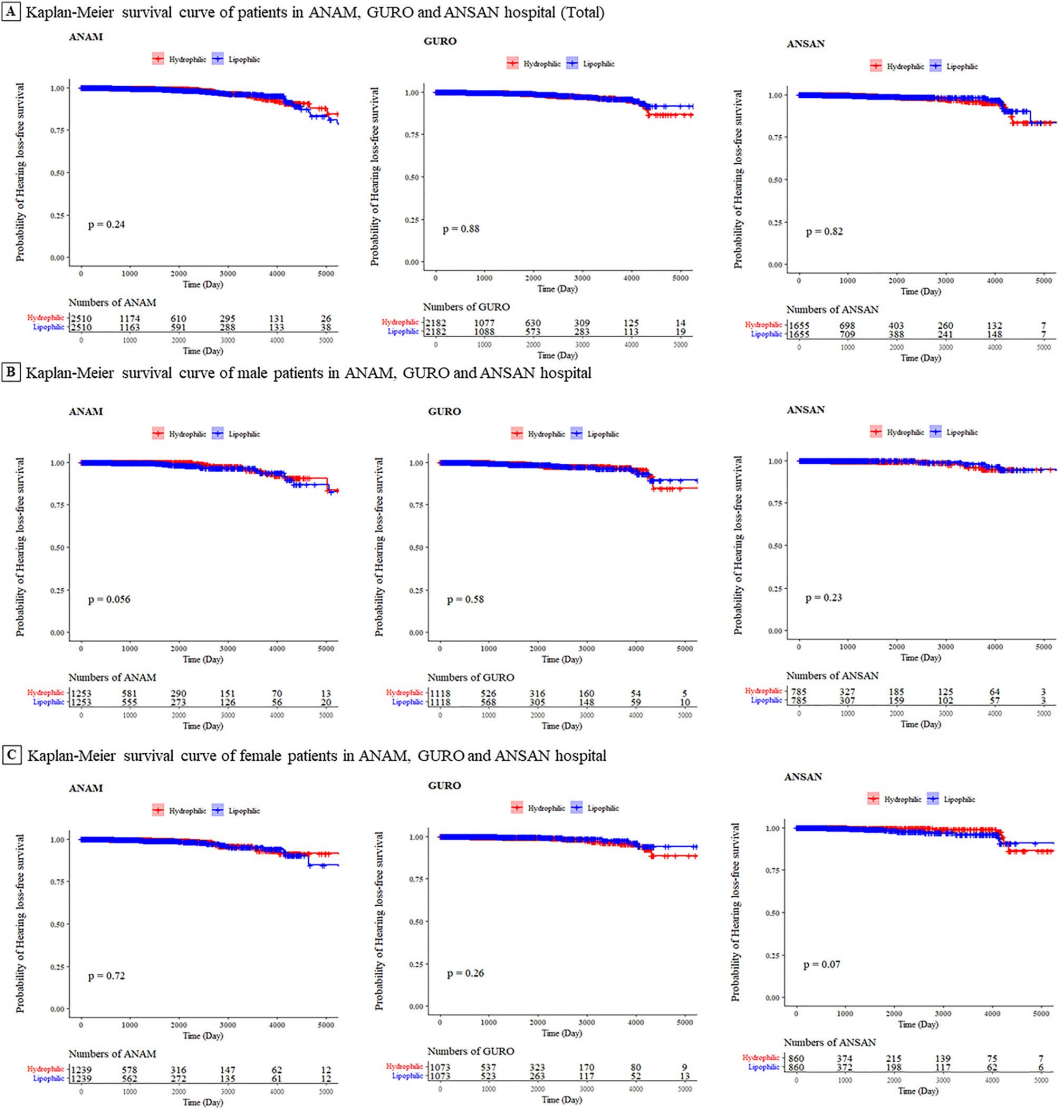
Kaplan–Meier survival curve of hearing loss comparing hydrophilic statin and lipophilic statin users. The Kaplan–Meier (KM) curves for overall survival comparing hydrophilic statin users and lipophilic statin users in the propensity score matching from the three hospitals. (**A**) shows the KM survival curve of the three hospitals; (**B**) represents the KM survival curve of hearing loss in male patients; (**C**) illustrates the case of female patients. P indicates the *p*-value of the Wilcoxon test of the two curves in each plot. Over 0.05 of *p*-value contents, there are no significant difference between two curves.

Figure 3 shows the Kaplan–Meier survival curve of hydrophilic and lipophilic statin users from all three hospitals (Fig. 3A for all patients, Fig. 3B for male patients, and Fig. 3C for female patients). When comparing each curve, no significant differences were found in the use of statins between the three hospitals.

**Figure 4:**
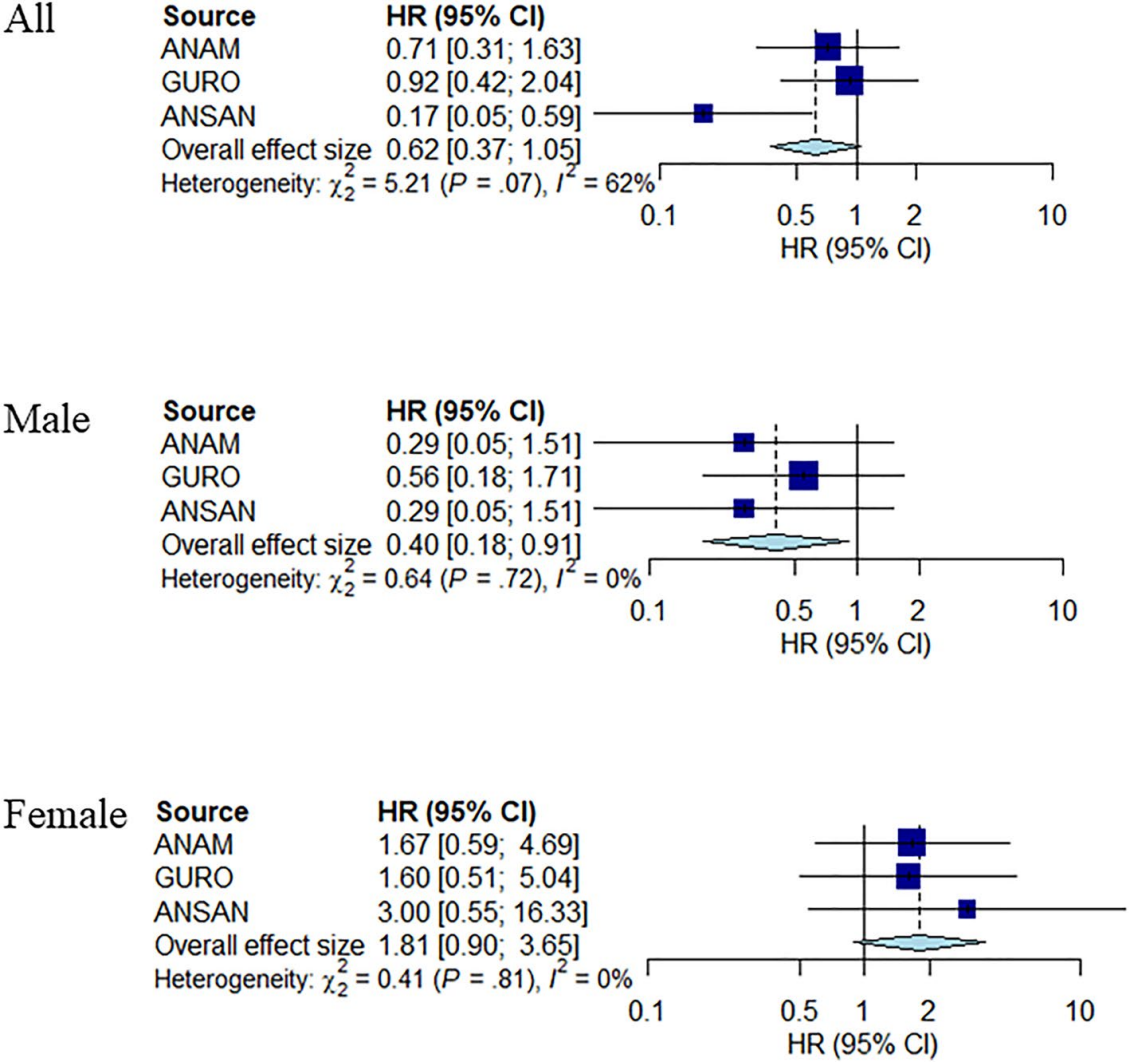
Forest plot of using hydrophilic and lipophilic statins in hearing loss for each hospital. Forest plots showing the hazard ratio (HR) and 95% confidence interval (CI) for hearing loss for the three hospitals. Overall effect sizes are estimated using a random-effect model. The size of the blue box represents the weight of the study. The horizontal line shows an error bar that indicates 95% CIs.

Nevertheless, the summary of the HRs from the three hospitals demonstrates interesting results. In Fig. 4, three forest plots represent the HR of hearing loss in each hospital, stratified by sex, estimated using Cox proportional regression. Precisely, the HR of all patient groups from the Anam (HR, 0.71, [95% CI 0.31–1.63]), Guro (HR, 0.92, [95% CI 0.42–2.04]), and Ansan (HR, 0.17, [95% CI 0.05–0.59]) shows that the risk of hearing loss among hydrophilic statin users was less than that among lipophilic statin users. However, it is not statistically significant considering that the CIs bordered 1. Overall the effect size is the summary of the effect size executed by the meta-analysis (HR, 0.62, [95% CI 0.37–1.05]) and demonstrates the same result for all patients in the three hospitals.

However, the HR of the male patient groups from the Anam (HR, 0.29, [95% CI 0.05–1.51]), Guro (HR, 0.56, [95% CI 0.18–1.71]), and Ansan (HR, 0.29, [95% CI 0.05–1.51]) hospitals still indicates that the risk of hearing loss in hydrophilic statin users was less compared to that in lipophilic statin users. However, the overall effect size (HR, 0.40, [95% CI 0.18–0.91]) was statistically significant.

In addition to the overall effect size (HR, 1.81, [95% CI 0.90–3.65]), the HR of female patient groups from the Anam (HR, 1.67, [95% CI 0.59–4.69]), Guro (HR, 1.60, [95% CI 0.51–5.04]), and Ansan (HR, 3.00, [95% CI 0.90–3.65]) hospitals showed a higher risk of hearing loss in hydrophilic statin users than that in lipophilic statin users. Although these results differ from the previous ones, they were not statistically significant.

### Negative control analysis

Figure S1 shows the negative control effect size from three hospitals based on sex. The concept ID of the negative outcomes is described in Table S2. As shown in Fig. S1, majority of the negative controls have a CI that includes an HR of 1, indicating that the analysis does not have a systematic error.

## Discussion

Statins belong to a class of drugs that are widely used for treating hyperlipidemia and preventing heart attacks and strokes. They work by inhibiting HMG-CoA reductase to varying degrees. Drugs classified as statins share common mechanisms of action while displaying distinct molecular structures and varying degrees of lipophilicity or hydrophilicity. The variation in their chemical structure contributes to factors such as enzyme binding affinity, drug efficacy, tissue selectivity, bioavailability, degradation mechanism, absorption mechanism, and elimination half-life^24^.

Previous research on the association between statin use and sudden sensorineural hearing loss has yielded conflicting results^25,26^. Studies on statins and sensorineural hearing loss have generally found a reduced risk of hearing impairment^27–29^. In a study using national health screening data, the odds ratio of hearing impairment was lower in the statin group, with a more significant effect observed in the hydrophilic statin group^9^.

Since statins are not clinically used to treat hearing impairment, it is difficult to conduct a meta-analysis using large-scale data. However, CDM models enable accurate analysis by creating uniformity in large-scale data and allowing reproducibility. The effectiveness of CDM has been demonstrated in animal experiments and retrospective studies in patients. The CDM cohort study is a correlation analysis of specific topics using a model that standardizes large clinical data from each hospital in terms of structure and meaning. Organizing CDMs preserve the original data from a source and allows maximum adaptability. Fully organized data models are easy to utilize because all rough codes are mapped to a medical composition. The adaptive rules system expands a database of easily adaptable, reusable measures to maintain adaptability, facilitate analysis, and ensure study-specific transparency^13^. Therefore, we conducted the first large-scale CDM cohort study to investigate the effect of hydrophilic and lipophilic statins on hearing loss in patients with hyperlipidemia. Our study found that hydrophilic statins had a protective effect on hearing impairment compared to that of lipophilic statins in men. Homogenization was conducted because this study included a large patient group from three hospitals, and the patients were divided into two groups: hydrophilic and lipophilic statin users. Moreover, the variations of the patient group were corrected through covariate balancing and PS matching.

In this study, the protective effect of hydrophilic statins over lipophilic statins against hearing impairment was only significant in males, which is consistent with the results of previous studies (from similar settings using healthcare cohort data) that have also shown that the protective effect of statins is more prominent in older and male populations more susceptible to cardiovascular events^9^. The protective effect of statins may be stronger in men due to a higher rate of risk factors for cardiovascular diseases such as obesity, alcohol use, or smoking^30^. Additionally, the more careful approach to health of women may cause increased suspicions regarding the need for statins or more distresses about their potential side effects. Therefore, men have taken statins for a relatively longer duration than have women^12,30,31^. Hydrophilic statins may have fewer side effects compared to those of lipophilic statins due to their lower tissue uptake and reduced dependence on cytochrome P450 metabolism^24^. In addition, a previous study that included patients with atherosclerosis reported superior anti-inflammatory effects (with higher levels of adiponectin and lower levels of C-reactive protein) of hydrophilic statins compared to the effects of lipophilic statins^32^.

This study has several limitations. First, because this was a code-based study, we might have determined hearing loss only in the context of codes. Conducting a thorough qualitative analysis would have been ideal. Furthermore, we did not conduct an in-depth analysis of the correlation between the duration of statin administration and the degree of hearing loss. Moreover, we did not consider the degree of hyperlipidemia related to coding. Future studies should consider these limitations, in addition to including a control group (with no statin intake).

In conclusion, our large-scale, multicenter study provides evidence supporting the protective effect of hydrophilic statins on hearing impairment. In particular, our findings indicate that men in the hydrophilic statin group exhibited a lower risk of hearing impairment than did those in the lipophilic statin group. This research expands our understanding of the differential impacts of hydrophilic and lipophilic statins and highlights their potential in mitigating hearing loss in patients with hyperlipidemia.

## Supplementary Information

Supplementary Information 1.

## Data Availability

The datasets generated and/or analyzed during the current study are not publicly available due to the legal restrictions of South Korea but are available from the corresponding author on reasonable request.
