# Supplementary material for: Hydrophilic and lipophilic statin use and risk of hearing loss in hyperlipidemia using a Common Data Model: multicenter cohort study
Source: Sci Rep. 2023 Jul 31;13:12373. doi: 10.1038/s41598-023-39316-x (PMC10390480; doi:10.1038/s41598-023-39316-x)

**Supplementary Information**

**Supplementary Table S1.** List of Concept ID and definitions

**Supplementary Table S2.** Number of statin cases in the study participants before and after propensity score matching

**Supplementary Table S3.** Study participants’ selected baseline characteristics before and after propensity score matching by sex

**Supplementary Figure S1.** Negative control effect size of three hospitals

**Supplementary Figure S2.** The propensity score distribution and covariate balancing before and after matching of male and female patients in three hospitals

This supplemental information has been provided by the authors to give readers additional information about their work.

| **Supplementary Table S1.** List of concept ID and definitions | |
| --- | --- |
| **Definition** | **Concept ID** |
| **Hyperlipidemia** | 432867, 438720, 4029305, 4120314 |
| **Hearing loss** | 374053, 374366, 374367, 375826, 377888, 377889, 379832, 381312, 433495, 436421, 440422, 444291, 4110815, 4232398, 37110393 |
| **Use of Hydrophilic statin** | 2061312, 2063242, 2063246, 2063250, 2063252, 2063256, 2063260, 2063264, 2064498, 2064532, 2064553, 2064586, 2065482, 2065486, 2065490, 2066020, 2066021, 2066023, 2066024, 19022104, 36026843, 36026845, 36026847, 40165245, 40165253, 40165261, 40175390, 40175394, 40175400, 42968976, 42968981, 42968990, 42968999, 42969008, 42969022, 42969031, 42969040, 42969049, 42969058, 42969067, 42969082, 42969085, 42969088, 42969091, 42969094, 42969097, 42969102, 42969106, 42969109, 42969112, 42969115, 42969118, 42969123, 42969126, 42969129, 42969132, 42969135, 42969138, 42969141, 42969149, 42969152, 42969154, 42969157, 42969162, 42969165, 42969168 |
| **Use of Lipophilic statin** | 1332494, 1332495, 1332497, 1332499, 1539407, 1539411, 1539469, 1545959, 1545996, 1545997, 2054845, 2057647, 2057655, 2057662, 2057670, 2057677, 19019116, 19077498, 19077499, 19112569, 19122209, 19123592, 40165638, 40165642, 40165646, 42932538, 42932541, 42932544, 42932547, 42936873, 42972631, 42972634, 42972637, 42972640, 43527029, 43527032, 44506638, 44506641 |
| **Diabetes mellitus** | 201254, 201826, 4008576, 4058243 |
| **Hypertensive disorder**  **(Essential hypertension)** | 319826, 320128, 4028741, 4071202 |
| **Aminoglycoside** | 902807, 1594686, 1836193, 3035509, 19017588, 19057566, 21059019, 21156684, 21156995, 21166988, 35603927, 35604082, 35604084, 35604087, 41242987, 41398251, 42919634, 42919699, 42921825, 42921978, 42939506 |
| **Smoker** | 4041306 |
| **Acute bronchitis** | 260139 |
| **Gastro-esophageal reflux disease with esophagitis** | 30437 |
| **Gingival and periodontal disease** | 132344 |
| **Spinal stenosis** | 77079 |

| **Supplementary Table S2.** Number of statin cases in the study participants before and after propensity score matching. | | | | | | |
| --- | --- | --- | --- | --- | --- | --- |
| **Center** | **PSM**${}^{\mathbf{a}}$ | **Case** | **Subjects**${}^{\mathbf{b}}$ | **Hearing loss** | **Incidence Rate**${}^{\mathbf{c}}$ | **Time at Risk** |
| ANAM | Before | Hydrophilic statin users | 5,605 | 55 | 3.09 | 17776.49 |
|  |  | Lipophilic statin users | 8,309 | 104 | 2.90 | 35864.79 |
|  | After | Hydrophilic statin users | 2,510 | 31 | 3.33 | 9299.56 |
|  |  | Lipophilic statin users | 2,510 | 36 | 3.89 | 9239.83 |
| GURO | Before | Hydrophilic statin users | 4,849 | 49 | 2.92 | 16788.36 |
|  |  | Lipophilic statin users | 7,439 | 93 | 2.74 | 33943.42 |
|  | After | Hydrophilic statin users | 2,182 | 27 | 3.12 | 8663.87 |
|  |  | Lipophilic statin users | 2,182 | 21 | 2.48 | 8450.56 |
| ANSAN | Before | Hydrophilic statin users | 3,122 | 25 | 2.75 | 9077.96 |
|  |  | Lipophilic statin users | 7,566 | 112 | 3.70 | 30231.44 |
|  | After | Hydrophilic statin users | 1,655 | 20 | 3.30 | 6061.24 |
|  |  | Lipophilic statin users | 1,655 | 25 | 3.33 | 6002.93 |
| ANAM  (Male) | Before | Hydrophilic statin users | 3,069 | 31 | 3.27 | 9491.00 |
|  |  | Lipophilic statin users | 3,834 | 43 | 2.72 | 15814.79 |
|  | After | Hydrophilic statin users | 1,253 | 15 | 3.24 | 4621.70 |
|  |  | Lipophilic statin users | 1,253 | 17 | 3.87 | 4387.26 |
| GURO  (Male) | Before | Hydrophilic statin users | 2,870 | 27 | 2.73 | 9891.05 |
|  |  | Lipophilic statin users | 3,502 | 42 | 2.61 | 16061.87 |
|  | After | Hydrophilic statin users | 1,118 | 13 | 2.99 | 4339.48 |
|  |  | Lipophilic statin users | 1,118 | 14 | 3.16 | 4425.54 |
| ANSAN  (Male) | Before | Hydrophilic statin users | 1,644 | 12 | 2.59 | 4618.12 |
|  |  | Lipophilic statin users | 3,907 | 41 | 2.68 | 15264.95 |
|  | After | Hydrophilic statin users | 785 | 7 | 2.45 | 2851.37 |
|  |  | Lipophilic statin users | 785 | 6 | 2.26 | 2650.88 |
| ANAM  (Female) | Before | Hydrophilic statin users | 2,536 | 24 | 2.89 | 8284.52 |
|  |  | Lipophilic statin users | 4,475 | 61 | 3.04 | 20049.21 |
|  | After | Hydrophilic statin users | 1,239 | 14 | 3.03 | 4622.79 |
|  |  | Lipophilic statin users | 1,239 | 16 | 3.60 | 4438.92 |
| GURO  (Female) | Before | Hydrophilic statin users | 1,979 | 22 | 3.19 | 6897.32 |
|  |  | Lipophilic statin users | 3,937 | 51 | 2.85 | 17881.55 |
|  | After | Hydrophilic statin users | 1,073 | 13 | 2.96 | 4394.88 |
|  |  | Lipophilic statin users | 1,073 | 6 | 1.49 | 4035.49 |
| ANSAN  (Female) | Before | Hydrophilic statin users | 1,491 | 7 | 1.56 | 4484.76 |
|  |  | Lipophilic statin users | 3,679 | 40 | 2.66 | 15042.28 |
|  | After | Hydrophilic statin users | 860 | 6 | 1.86 | 3215.92 |
|  |  | Lipophilic statin users | 860 | 10 | 3.23 | 3092.43 |

${}^{a}$ PSM; Propensity score matching

${}^{b}$ ANAM; Exclude participants who have the outcome prior to the risk window start (n=70) and do not have at least 1 day at risk (n=107)

GURO; Exclude participants who have the outcome prior to the risk window start (n=53) and do not have at least 1 day at risk (n=103)

ANSAN; Exclude participants who have the outcome prior to the risk window start (n=52) and do not have at least 1 day at risk (n=107)

ANAM (Male); Exclude participants who have the outcome prior to the risk window start (n=38) and do not have at least 1 day at risk (n=52)

GURO (Male); Exclude participants who have the outcome prior to the risk window start (n=31) and do not have at least 1 day at risk (n=51)

ANSAN (Male); Exclude participants who have the outcome prior to the risk window start (n=17) and do not have at least 1 day at risk (n=44)

ANAM (Female); Exclude participants who have the outcome prior to the risk window start (n=32) and do not have at least 1 day at risk (n=55)

GURO (Female); Exclude participants who have the outcome prior to the risk window start (n=22) and do not have at least 1 day at risk (n=52)

ANSAN (Female); Exclude participants who have the outcome prior to the risk window start (n=22) and do not have at least 1 day at risk (n=43)

${}^{c}$ Incidence rate (IR) per 1,000 person-years

**Supplementary Table S3.** Study participants’ selected baseline characteristics before and after propensity score matching by sex

| **Characteristic** | **ANAM (Male)** | | | | | |
| --- | --- | --- | --- | --- | --- | --- |
|  | **Before Matching** | | | **After Matching** | | |
|  | **Hydrophilic users (n = 3,107)** | **Lipophilic users**  **(n = 3,886)** | **Standard Difference** | **Hydrophilic users  (n = 1,253)** | **Lipophilic users (n = 1,253)** | **Standard Difference** |
|  | **No. (%)** | **No. (%)** |  | **No. (%)** | **No. (%)** |  |
| Age |  |  |  |  |  |  |
| 40 – 44 | 244 (7.8) | 306 (7.8) | 0.00 | 114 (9.1) | 109 (8.7) | 0.01 |
| 45 – 49 | 322 (10.4) | 423 (10.8) | –0.02 | 137 (10.9) | 149 (11.9) | –0.03 |
| 50 – 54 | 458 (14.7) | 533 (13.7) | 0.03 | 187 (14.9) | 180 (14.4) | 0.02 |
| 55 – 59 | 510 (16.4) | 568 (14.6) | 0.05 | 200 (15.9) | 198 (15.8) | 0.00 |
| 60 – 64 | 533 (17.2) | 620 (15.9) | 0.03 | 209 (16.7) | 208 (16.6) | 0.00 |
| 65 – 69 | 431 (13.9) | 537 (13.8) | 0.00 | 155 (12.4) | 154 (12.3) | 0.00 |
| 70 – 74 | 318 (10.2) | 486 (12.5) | –0.07 | 136 (10.8) | 135 (10.8) | 0.00 |
| 75 – 79 | 247 (7.9) | 364 (9.4) | –0.05 | 94 (7.5) | 107 (8.5) | –0.04 |
| ≥ 80 | 44 (1.4) | 49 (1.3) | 0.01 | 21 (1.7) | 13 (1.0) | 0.06 |
| Diabetes mellitus | 612 (19.7) | 935 (24.0) | –0.11 | 275 (21.9) | 259 (20.7) | 0.03 |
| Hypertensive disorder | 1474 (47.4) | 1677 (43.2) | 0.09 | 506 (40.4) | 549 (43.8) | –0.07 |
| Smoker | 584 (18.8) | 939 (24.2) | -0.13 | 230 (18.3) | 215 (17.2) | 0.03 |

| Characteristic | **GURO (Male)** | | | | | |
| --- | --- | --- | --- | --- | --- | --- |
|  | **Before Matching** | | | **After Matching** | | |
|  | **Hydrophilic users (n = 2,901)** | **Lipophilic users**  **(n = 3,553)** | **Standard Difference** | **Hydrophilic users  (n = 1,118)** | **Lipophilic users (n = 1,118)** | **Standard Difference** |
|  | **No. (%)** | **No. (%)** |  | **No. (%)** | **No. (%)** |  |
| Age |  |  |  |  |  |  |
| 40 – 44 | 211 (8.2) | 270 (7.6) | –0.01 | 92 (7.8) | 88 (7.9) | 0.01 |
| 45 – 49 | 308 (10.9) | 362 (10.2) | 0.01 | 122 (11.0) | 123 (11.0) | 0.00 |
| 50 – 54 | 434 (15.8) | 530 (14.9) | 0.00 | 177 (15.8) | 181 (16.2) | –0.01 |
| 55 – 59 | 508 (17.7) | 626 (17.6) | 0.00 | 198 (17.7) | 203 (18.1) | –0.01 |
| 60 – 64 | 563 (17.3) | 580 (16.3) | 0.08 | 194 (17.3) | 179 (16.0) | 0.04 |
| 65 – 69 | 402 (14.1) | 528 (14.9) | –0.03 | 158 (14.1) | 165 (14.7) | –0.02 |
| 70 – 74 | 263 (9.7) | 369 (11.1) | –0.07 | 109 (9.7) | 113 (10.1) | –0.01 |
| 75 – 79 | 185 (5.1) | 225 (6.3) | 0.00 | 57 (5.1) | 56 (5.0) | 0.00 |
| ≥ 80 | 27 (1.0) | 36 (1.0) | –0.01 | 11 (1.0) | 10 (0.9) | 0.01 |
| Diabetes mellitus | 629 (22.4) | 923 (25.9) | –0.10 | 251 (22.4) | 244 (21.8) | 0.02 |
| Hypertensive disorder | 1270 (44.2) | 1754 (49.4) | –0.11 | 494 (44.2) | 464 (41.5) | 0.05 |
| Smoker | 660 (12.3) | 488 (13.7) | 0.24 | 137 (12.3) | 157 (14.0) | –0.05 |

| **Characteristic** | **ANSAN (Male)** | | | | | |
| --- | --- | --- | --- | --- | --- | --- |
|  | **Before Matching** | | | **After Matching** | | |
|  | **Hydrophilic users (n = 1,661)** | **Lipophilic users**  **(n = 3,951)** | **Standard Difference** | **Hydrophilic users  (n = 785)** | **Lipophilic users (n = 785)** | **Standard Difference** |
|  | **No. (%)** | **No. (%)** |  | **No. (%)** | **No. (%)** |  |
| Age |  |  |  |  |  |  |
| 40 – 44 | 171 (10.3) | 499 (12.6) | –0.07 | 89 (11.3) | 92 (11.7) | –0.01 |
| 45 – 49 | 255 (15.4) | 630 (15.9) | –0.02 | 116 (14.8) | 104 (13.2) | 0.04 |
| 50 – 54 | 344 (20.7) | 707 (17.9) | 0.07 | 167 (21.3) | 142 (18.1) | 0.08 |
| 55 – 59 | 307 (18.5) | 714 (18.1) | 0.01 | 127 (16.2) | 140 (17.8) | –0.04 |
| 60 – 64 | 244 (14.7) | 535 (13.5) | 0.03 | 109 (13.9) | 135 (17.2) | –0.09 |
| 65 – 69 | 166 (10) | 333 (8.4) | 0.05 | 79 (10.1) | 72 (9.2) | 0.03 |
| 70 – 74 | 101 (6.1) | 279 (7.1) | –0.04 | 57 (7.3) | 51 (6.5) | 0.03 |
| 75 – 79 | 64 (3.9) | 223 (5.6) | –0.08 | 38 (4.8) | 44 (5.6) | –0.03 |
| ≥ 80 | 9 (0.5) | 31 (0.8) | –0.03 | 3 (0.4) | 5 (0.6) | –0.04 |
| Diabetes mellitus | 431 (25.9) | 861 (21.8) | 0.10 | 227 (28.9) | 231 (29.4) | –0.01 |
| Hypertensive disorder | 802 (48.3) | 1703 (43.1) | 0.10 | 358 (45.6) | 396 (50.4) | –0.10 |
| Smoker | 240 (14.4) | 687 (17.4) | –0.08 | 109 (13.9) | 91 (11.6) | 0.07 |

| **Characteristic** | **ANAM (Female)** | | | | | |
| --- | --- | --- | --- | --- | --- | --- |
|  | **Before Matching** | | | **After Matching** | | |
|  | **Hydrophilic users (n = 2,568)** | **Lipophilic users**  **(n = 4,530)** | **Standard Difference** | **Hydrophilic users  (n = 1,239)** | **Lipophilic users (n = 1,239)** | **Standard Difference** |
|  | **No. (%)** | **No. (%)** |  | **No. (%)** | **No. (%)** |  |
| Age |  |  |  |  |  |  |
| 40 – 44 | 72 (2.8) | 120 (2.6) | 0.01 | 35 (3.0) | 37 (3.0) | –0.01 |
| 45 – 49 | 136 (5.3) | 232 (5.1) | 0.01 | 74 (6.0) | 73 (5.9) | 0.00 |
| 50 – 54 | 328 (12.8) | 567 (12.5) | 0.01 | 164 (13.2) | 160 (12.9) | 0.01 |
| 55 – 59 | 464 (18.1) | 803 (17.7) | 0.01 | 228 (18.4) | 225 (18.2) | 0.01 |
| 60 – 64 | 454 (17.7) | 868 (19.2) | –0.04 | 219 (17.7) | 245 (19.8) | –0.05 |
| 65 – 69 | 425 (16.5) | 751 (16.6) | 0.00 | 198 (16.0) | 189 (15.2) | 0.02 |
| 70 – 74 | 351 (13.7) | 621 (13.7) | 0.00 | 159 (12.8) | 156 (12.6) | 0.01 |
| 75 – 79 | 280 (10.9) | 497 (11.0) | 0.00 | 132 (10.6) | 133 (10.7) | 0.00 |
| ≥ 80 | 58 (1.3) | 71 (1.6) | 0.05 | 30 (2.4) | 21 (1.7) | 0.05 |
| Diabetes mellitus | 400 (15.6) | 930 (20.5) | –0.13 | 215 (17.3) | 204 (16.5) | 0.02 |
| Hypertensive disorder | 1102 (42.9) | 1781 (39.3) | 0.07 | 496 (40.0) | 501 (40.4) | –0.01 |
| Smoker | 429 (16.7) | 875 (19.3) | –0.07 | 199 (16.1) | 196 (15.8) | 0.01 |

| **Characteristic** | **GURO (Female)** | | | | | |
| --- | --- | --- | --- | --- | --- | --- |
|  | **Before Matching** | | | **After Matching** | | |
|  | **Hydrophilic users (n = 2,001)** | **Lipophilic users**  **(n = 3,989)** | **Standard Difference** | **Hydrophilic users  (n = 1,073)** | **Lipophilic users (n = 1,073)** | **Standard Difference** |
|  | **No. (%)** | **No. (%)** |  | **No. (%)** | **No. (%)** |  |
| Age |  |  |  |  |  |  |
| 40 – 44 | 68 (3.4) | 118 (2.9) | 0.03 | 37 (3.4) | 38 (3.5) | –0.01 |
| 45 – 49 | 126 (6.3) | 235 (5.9) | 0.02 | 74 (6.9) | 68 (6.3) | 0.02 |
| 50 – 54 | 242 (12.1) | 499 (12.5) | –0.01 | 127 (11.8) | 123 (11.5) | 0.01 |
| 55 – 59 | 375 (18.7) | 716 (17.9) | 0.02 | 205 (19.1) | 196 (18.3) | 0.02 |
| 60 – 64 | 389 (19.4) | 794 (19.9) | –0.01 | 206 (19.2) | 195 (18.2) | 0.03 |
| 65 – 69 | 317 (15.8) | 636 (15.9) | 0.00 | 176 (16.4) | 170 (15.8) | 0.02 |
| 70 – 74 | 254 (12.7) | 538 (13.5) | –0.02 | 142 (13.2) | 156 (14.5) | –0.04 |
| 75 – 79 | 198 (9.9) | 400 (10.0) | 0.00 | 93 (8.7) | 110 (10.3) | –0.05 |
| ≥ 80 | 32 (1.60 | 53 (1.3) | 0.02 | 13 (1.2) | 17 (1.6) | –0.03 |
| Diabetes mellitus | 322 (16.1) | 867 (21.7) | –0.14 | 199 (18.5) | 192 (17.9) | 0.02 |
| Hypertensive disorder | 845 (42.2) | 1766 (44.3) | –0.04 | 438 (40.8) | 442 (41.2) | –0.01 |
| Smoker | 297 (14.8) | 479 (12.0) | 0.08 | 133 (12.4) | 142 (13.2) | –0.03 |

| **Characteristic** | **ANSAN (Female)** | | | | | |
| --- | --- | --- | --- | --- | --- | --- |
|  | **Before Matching** | | | **After Matching** | | |
|  | **Hydrophilic users (n = 1,513)** | **Lipophilic users**  **(n = 3,722)** | **Standard Difference** | **Hydrophilic users  (n = 860)** | **Lipophilic users (n = 860)** | **Standard Difference** |
|  | **No. (%)** | **No. (%)** |  | **No. (%)** | **No. (%)** |  |
| Age |  |  |  |  |  |  |
| 40 – 44 | 62 (4.1) | 201 (5.4) | –0.06 | 40 (4.7) | 48 (5.6) | –0.04 |
| 45 – 49 | 149 (9.8) | 345 (9.3) | 0.02 | 82 (9.5) | 65 (7.6) | 0.07 |
| 50 – 54 | 271 (17.9) | 606 (16.3) | 0.04 | 146 (17) | 138 (16) | 0.03 |
| 55 – 59 | 307 (20.3) | 683 (18.4) | 0.05 | 157 (18.3) | 167 (19.4) | –0.03 |
| 60 – 64 | 243 (16.1) | 607 (16.3) | –0.01 | 130 (15.1) | 154 (17.9) | –0.08 |
| 65 – 69 | 188 (12.4) | 478 (12.8) | –0.01 | 117 (13.6) | 118 (13.7) | 0.00 |
| 70 – 74 | 157 (10.4) | 395 (10.6) | –0.01 | 94 (10.9) | 88 (10.2) | 0.02 |
| 75 – 79 | 115 (7.6) | 356 (9.6) | –0.07 | 76 (8.8) | 77 (9) | 0.00 |
| ≥ 80 | 21 (1.4) | 51 (1.4) | 0.00 | 18 (2.1) | 5 (0.6) | 0.13 |
| Diabetes mellitus | 293 (19.4) | 800 (21.5) | –0.05 | 203 (23.6) | 206 (24) | –0.01 |
| Hypertensive disorder | 613 (40.5) | 1474 (39.6) | 0.02 | 392 (45.6) | 405 (47.1) | –0.03 |
| Smoker | 179 (11.8) | 671 (18) | –0.17 | 114 (13.3) | 96 (11.2) | 0.06 |

**Supplementary Figure S1.** Negative control effect size of three hospitals


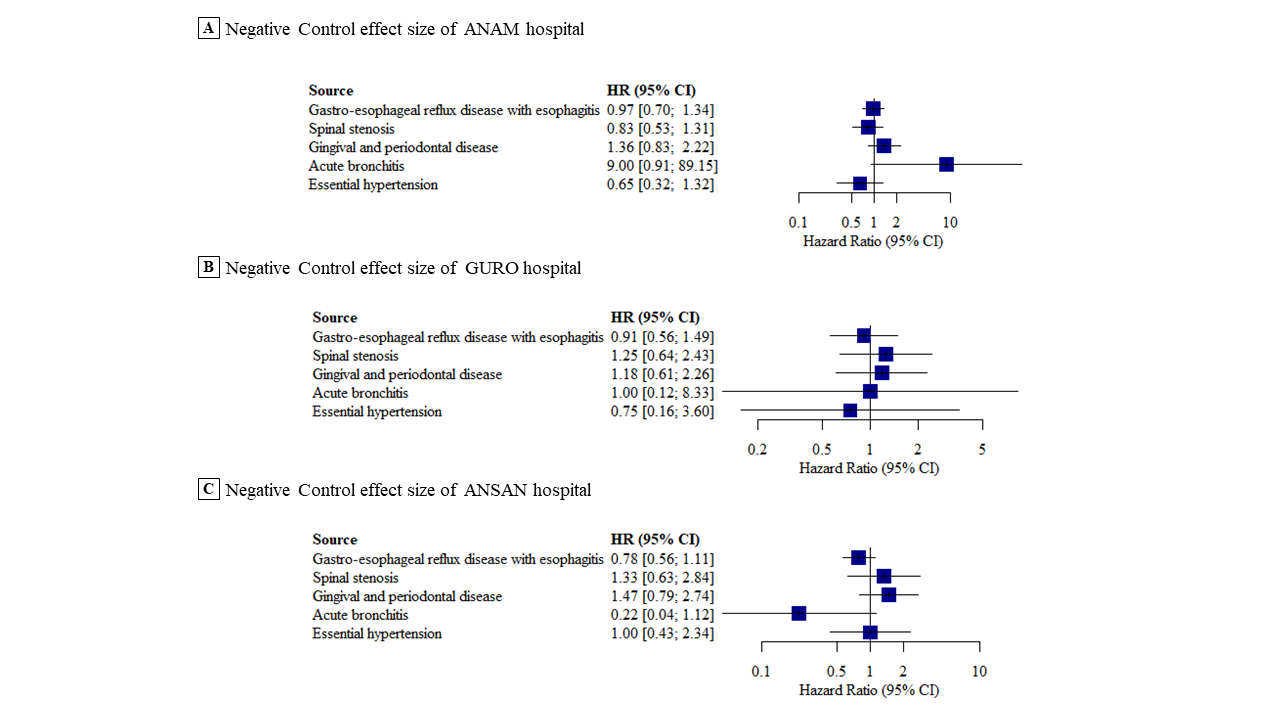

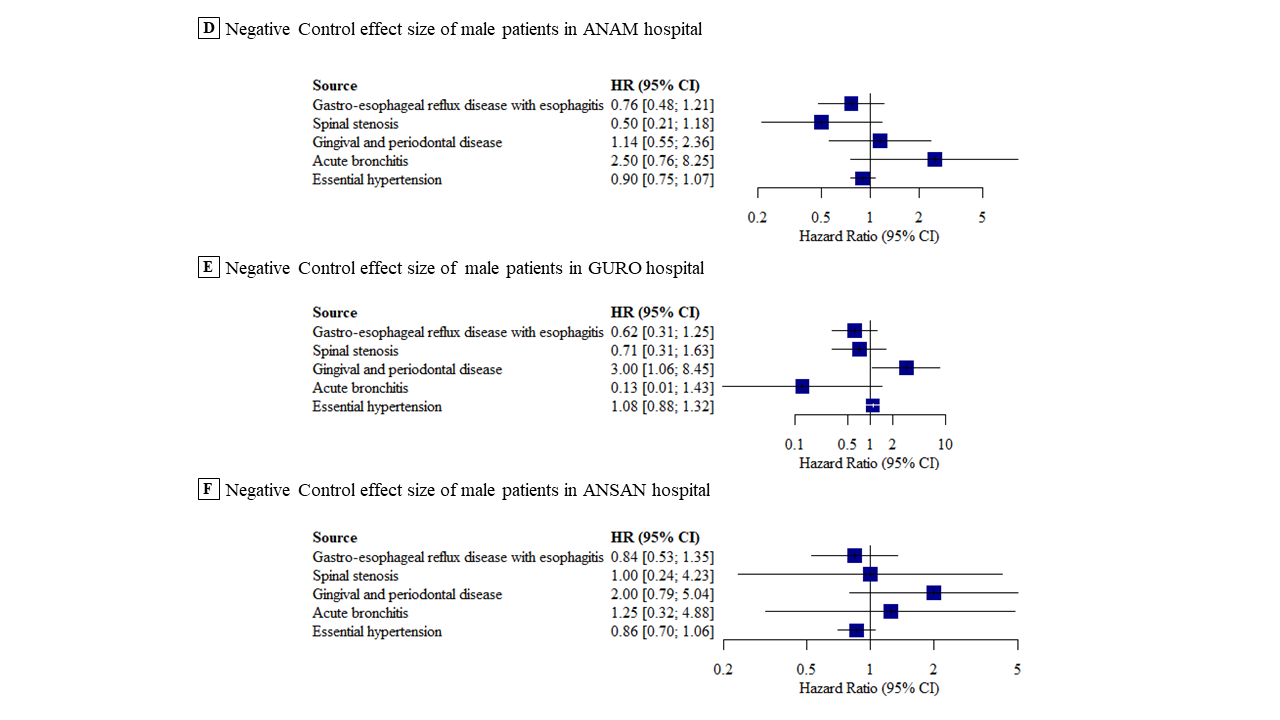


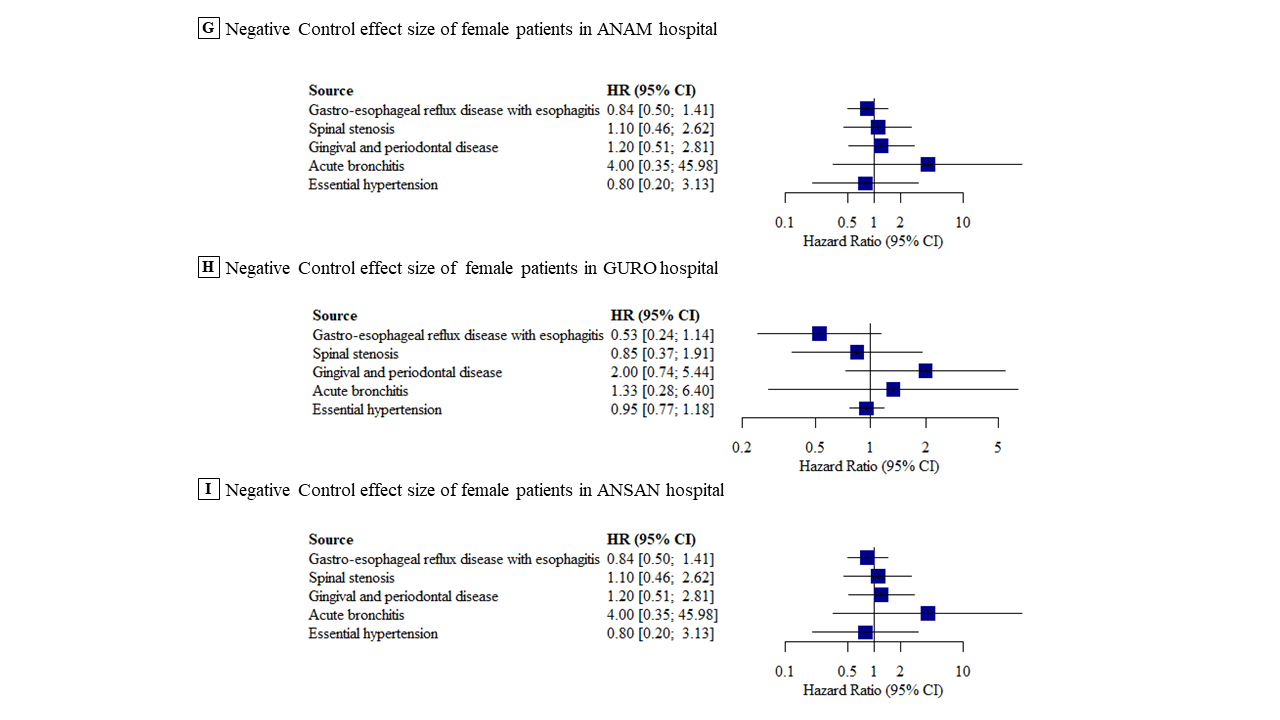


**Supplementary Figure S2.** The propensity score distribution and covariate balancing before and after matching of male and female patients in three hospitals


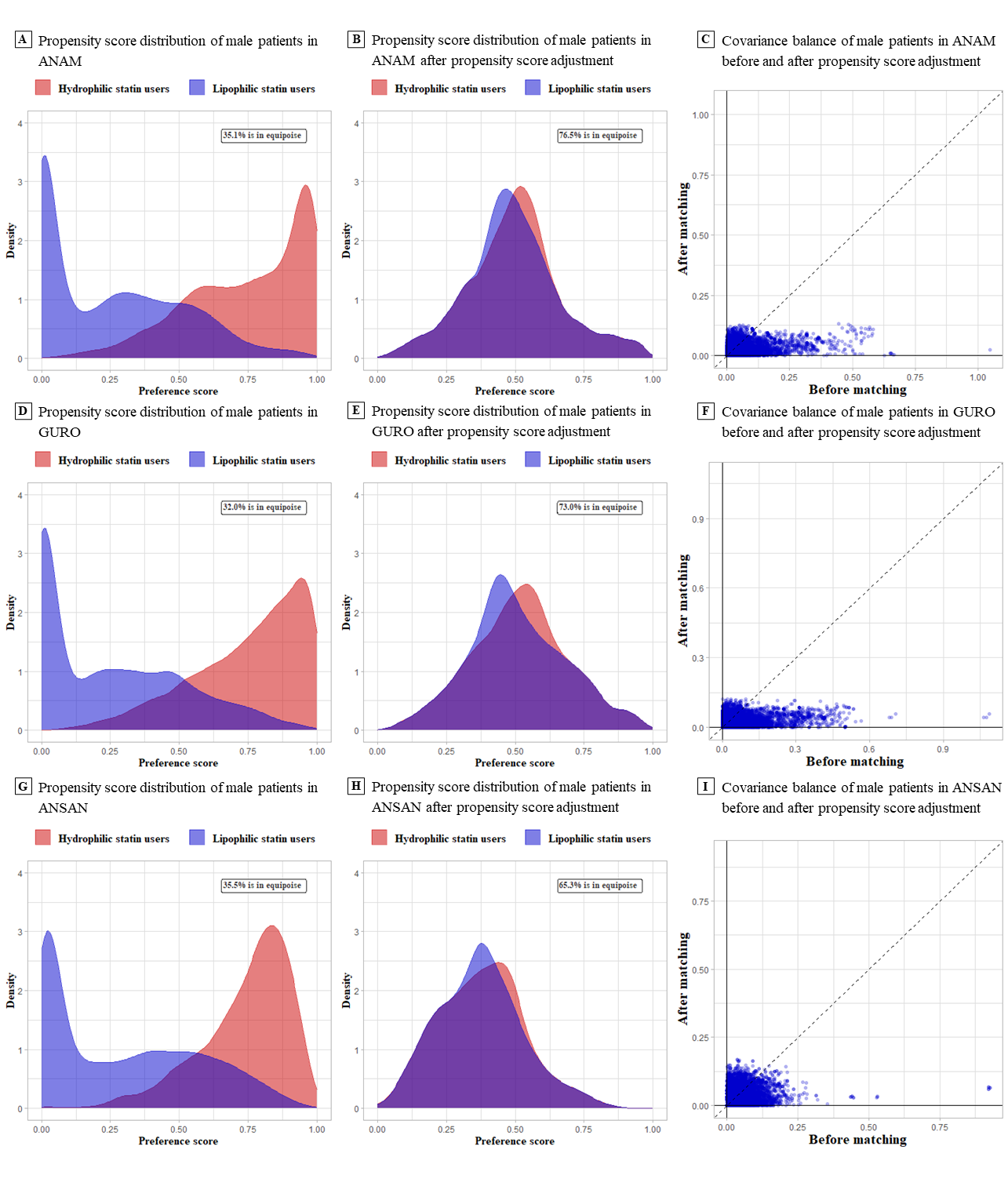


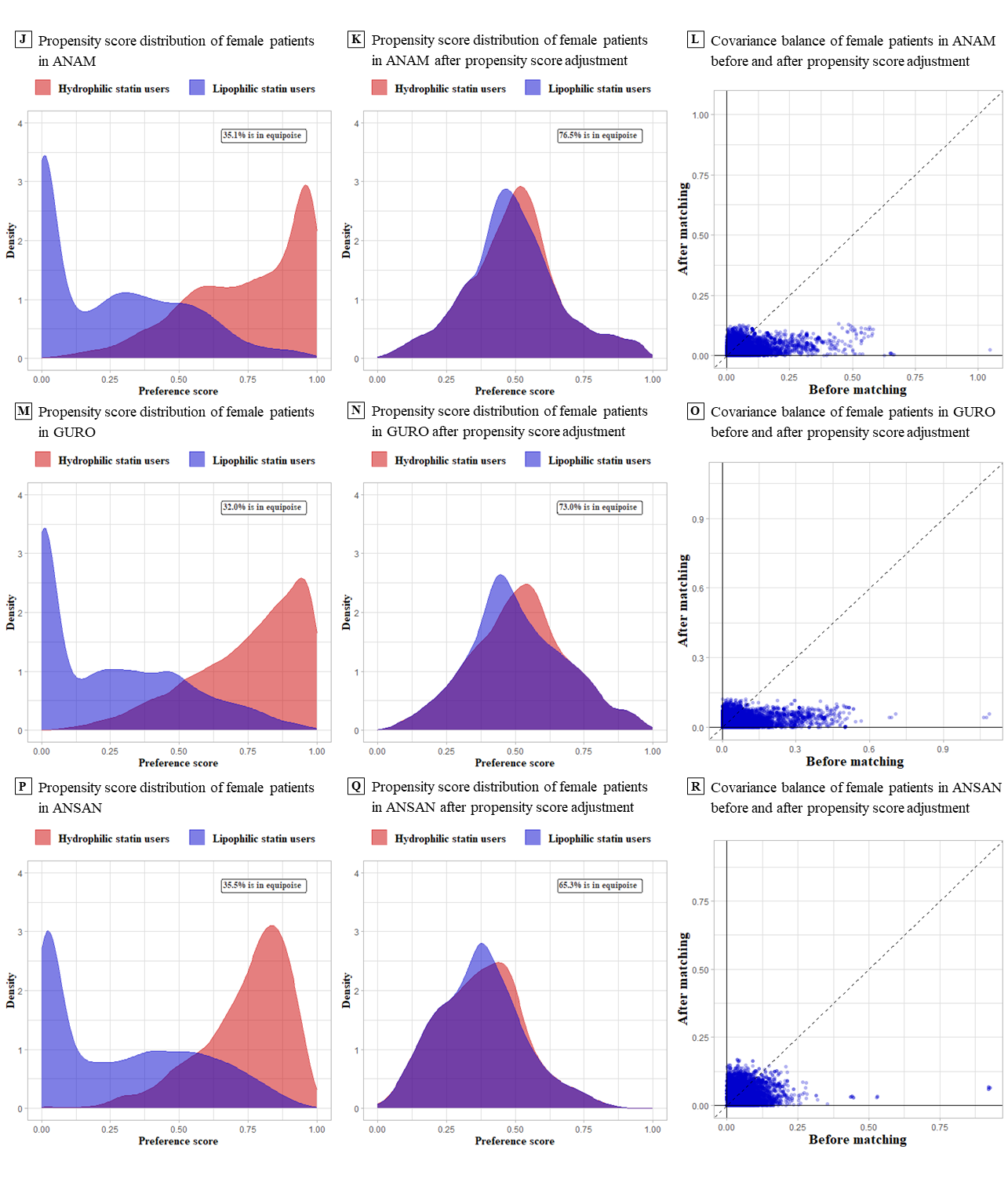

Supplement: Supplementary file 1 — Supplementary Information 1. [file 41598_2023_39316_MOESM1_ESM.docx]
